# Impact of the 2018 periodontal classification on diagnosis, treatment strategies, and assessment of prognosis by dental hygienists in Swedish general dental care

**DOI:** 10.2340/aos.v85.46574

**Published:** 2026-07-31

**Authors:** Emelie Stenberg, Nina Lundegren, Bengt Götrick, Aleksandar Milosavljevic

**Affiliations:** a Department of Oral Health, Faculty of Odontology, Malmö University, Malmö, Sweden; b Department of Oral Diagnostics, Faculty of Odontology, Malmö University, Malmö, Sweden; c Department of Orofacial Medicine, Faculty of Odontology, Malmö University, Malmö, Sweden; d Department of Periodontology, Faculty of Odontology, Malmö University, Malmö, Sweden

**Keywords:** Classification, general dentistry, periodontitis, treatment strategies

## Abstract

**Objective:**

To investigate the impact of the 2018 periodontal classification on diagnostic assessments, treatment strategies, and prognostic assessments made by dental hygienists in general dentistry, compared with the 1999 classification.

**Materials and Methods:**

A questionnaire comprising five patient cases was administered to dental hygienists in Sweden on two separate occasions. For each case, diagnoses, treatment plans, and prognostic assessments were proposed, first using the 1999 and subsequently the 2018 classification. Cases ranged from no marginal bone loss attributed to periodontitis and few (<10%) bleeding sites to marginal bone loss >30% and generalized inflammation (>30%). Answers from the two occasions were compared.

**Results:**

A total of 142 dental hygienists completed both questionnaires. Diagnostic assessments changed significantly in three of the five cases when using the 2018 classification (*p* < 0.05). In four cases, fewer treatment sessions were recommended, and more optimistic prognostic assessments were made when using the 2018 classification (*p* < 0.05).

**Conclusion:**

The 2018 classification resulted in greater variation in proposed diagnoses in cases without clear attachment loss and was associated with fewer recommended treatment sessions and more optimistic prognostic assessments. However, it appeared at least as effective as the 1999 classification in identifying cases with periodontitis.

## Introduction

In 2017, the American Academy of Periodontology (AAP) and the European Federation of Periodontology (EFP) developed a new classification for periodontitis, the 2018 AAP/EFP classification. In contrast to previous classifications, it defines the characteristics of a healthy periodontium and the characteristics of a stable periodontal status that need to be achieved for periodontal treatment to be considered successful. It also describes the severity of periodontitis in more detail (stages) and introduces the risk of disease progression as part of the classification (grade) [1–4].

Previous research on the use of the former periodontal classification, the 1999 AAP/CDC [5], uncovered variations in diagnosis, treatment planning, and prognostic assessment. Some survey studies found that clinicians varied in their diagnoses of patients with the same periodontal condition, which may place patients at risk of over- or undertreatment, depending on which clinician the patient visits [6–8]. The 1999 AAP/CDC classification was revised, among other aims, to minimize variation among clinicians and promote individually tailored treatment strategies [1, 3, 9].

Recent studies have evaluated diagnostic accuracy among groups of clinicians using the 2018 classification. In all categories (stage, grade, and extent), inter-rater agreement among periodontal experts was mostly moderate [10, 11] and among general dentists, low to moderate [12]. However, these studies have mostly focused on cases of moderate-to-severe periodontitis treated in a specialist/university setting, often excluding cases with mild or no periodontitis although the latter group represents more than 80% of the population [13, 14]. Studies focusing on clinicians in general dentistry who care for the large group of patients with no/mild periodontitis are lacking. Additionally, the effects of the 2018 classification on long-term prognosis have, to the best of our knowledge, not been evaluated.

In Swedish general dentistry, registered dental hygienists (RDHs) diagnose and treat patients with periodontitis in their daily practice [15]. They therefore represent a highly relevant professional group for examining the impact of periodontal classifications. The aim of this study was to investigate the effects of the 2018 periodontal classification on diagnostic assessments, treatment strategies, and long-term prognostic assessments in general dental care compared with the previous 1999 classification.

## Materials and methods

### Study design

This study employed a quasi-experimental within-subject pre- and post-test design. Reporting was conducted in accordance with the TREND checklist (Transparent Reporting of Evaluations with Nonrandomized Designs) [16] (Appendix 1). The Swedish Ethical Review Authority approved the study (daybook no. 2021-01433). All participants received written information about the study and signed a written consent form. They were informed that participation was voluntary, that they could discontinue their participation at any time without giving a reason, and that they were guaranteed confidentiality. The study was conducted between 2021-05-06 and 2022-10-05.

### Sample selection

All members of the Swedish Dental Hygienist Association (approximately 3,300 out of approx. 4,300 RDHs in Sweden) were invited to participate in the study. An a priori power analysis was not conducted. This was because the study had an exploratory approach, included a large number of outcome variables, and lacked prior data to estimate relevant effect sizes. The sample size was therefore based on the available population. Those who accepted the invitation were sent a short form to collect demographic data on age, gender, and workplace. The exclusion criteria were (1) not clinically active and (2) working in a specialist setting. Eligible participants were enrolled in the study.

### Questionnaire design and data collection

**Figure 1:**
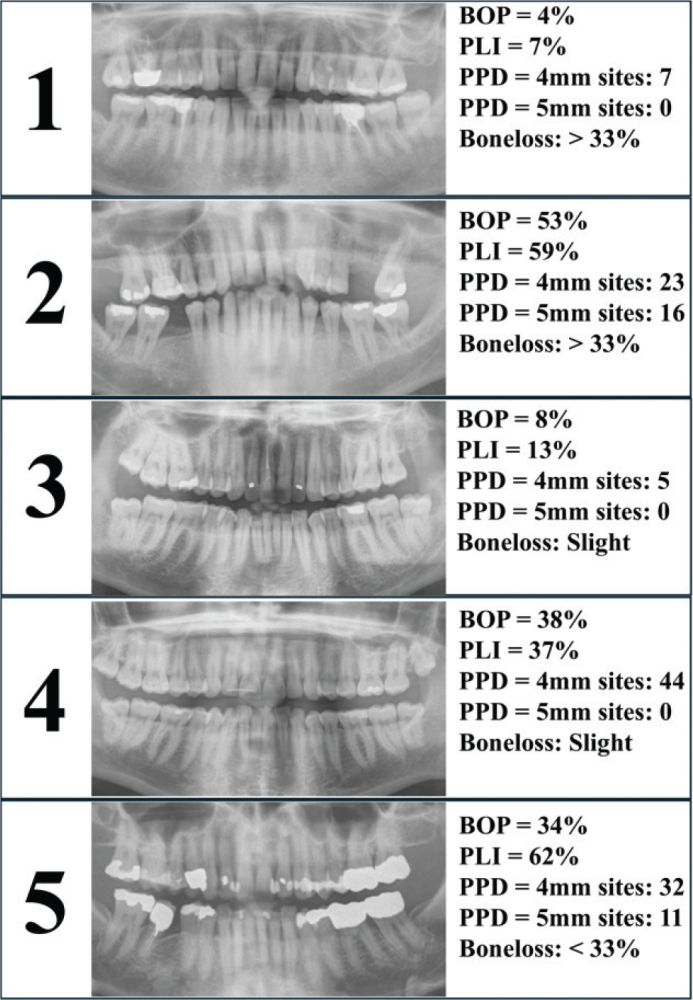
The five patient cases in the questionnaire. Periodontal status summaries. BOP: Bleeding on probing; PLI: Plaque score index; PPD: Probing pocket depth.

The enrolled participants received a questionnaire comprising five fictitious patient cases with varying periodontal status. The cases ranged from no marginal bone loss attributed to periodontitis and few (<10%) bleeding sites to marginal bone loss >30% and generalized inflammation (>30%) (Figure 1). All patients had the same anamnesis (male, 57 years old, systemically healthy, nonsmoker). Each case was described using a periodontal chart, a cropped panoramic image, four bitewing radiographs, and a radiological statement. The response sheet accompanying each case comprised five questions with predefined response options. For each case, participants were asked to propose a diagnosis and, when considered necessary, a treatment plan (comprising treatment measures and number of treatment sessions). They were also asked to indicate the need for supportive periodontal treatment (SPT) and suggest a prognostic assessment in the event of no treatment during the following 10 years (Table 1). The questionnaire was developed using web-based software, Artologik software for web (Sunet survey). It was based on a previously conducted study with the same fictitious patient cases [8] and additionally pilot tested with eight RDHs who were practicing in general dentistry and who were not otherwise involved in the study. The questionnaire was distributed on two occasions, the only difference being response options concerning diagnosis and which classification would be applied (1999 or 2018). To prevent multiple entries, the questionnaire was distributed via individual email invitations, allowing each recipient to respond only once. Efforts were made to minimize nonresponse by sending three reminders for the questionnaires.

**Table 1:** The questionnaire items and their responses according to the periodontal criteria used.

Questionnaire items	Response options	
	(1999 criteria)	(2018 criteria)
Gender Age Experience	M, F, nonbinary, or decline to state Years Years	M, F, nonbinary, or decline to state Years Years
Which diagnosis would you make?	- Localized gingivitis - Generalized gingivitis - Localized chronic periodontitis - Generalized chronic periodontitis - Not applicable - Other	- Periodontally healthy - Localized gingivitis - Generalized gingivitis - Periodontitis (stage and grade) - Stable periodontitis - Not applicable - Other
Does the patient require any periodontal treatment?	Yes/No	Yes/No
If yes, how many treatment sessions are required and which of the following treatments would you suggest? (Minimum: 0; maximum: 10 sessions for each item)	- Disease information (no. of occasions) - Oral hygiene instructions - Follow-up oral hygiene - Polishing - Supragingival scaling - Subgingival scaling - Periodontal surgery - Other	- Disease information (no. of occasions) - Oral hygiene instructions - Follow-up oral hygiene - Polishing - Supragingival scaling - Subgingival scaling - Periodontal surgery - Other
Is the patient in need of supportive treatment?	Yes/No	Yes/No
If the patient receives no treatment at all in the next 10 years, what changes should be expected in their periodontal status?	- Unchanged - Increased inflammation without bone loss - Increased bone loss - Tooth loss	- Unchanged - Increased inflammation without bone loss - Increased bone loss - Tooth loss

M: male; F: female; SPT: supportive periodontal therapy.

The questionnaire was administered twice: first, using the 1999 classification, and on the second occasion, using the 2018 classification.

### Occasion 1, evaluating the 1999 classification

On occasion 1, the questionnaire was accompanied by a short-form document instructing participants to use the 1999 classification when filling out the questionnaire. The short-form document included information regarding the available diagnostic categories in the 1999 classification. No education was given since the 1999 classification was well established in clinical practice.

### Occasion 2, evaluating the 2018 classification

On occasion 2, the questionnaire was accompanied by a short-form document, which prompted the participants to use the 2018 classification when completing the questionnaire. To ensure that participants had a basic understanding of the 2018 periodontal classification prior to completing the questionnaire on the second occasion, all participants completed a short education. This educational component was required because the 2018 classification was not generally implemented in Sweden at the time the study was conducted. Specifically, participants were given access to a pre-recorded lecture and were provided with a written summary describing the 2018 periodontal classification. They then participated in an online interactive workshop [17]. In addition, participants were able to view a schematic appendix, including a framework that describes the transition from periodontal health to gingivitis and further to periodontitis and a schematic overview of the classification (stage, grade, and extent) when filling out the questionnaire (Appendices 2–4).

### Data management and statistical analysis

Participants’ responses for each case concerning diagnosis, treatment strategy, and prognostic assessment were compared between the two occasions. During analysis, the proposed diagnoses were grouped: localized and generalized gingivitis were categorized as gingivitis; localized/generalized chronic periodontitis, periodontitis (stage and grade), and stable periodontitis were categorized as periodontitis. In the questionnaire on the first occasion, ‘healthy’ was not included as an answer option; instead, it was defined if the participants chose the option ‘other’ and freely wrote ‘healthy’. In the questionnaire on the second occasion, ‘healthy’ was included as a predefined option (Table 1).

Categorical variables, such as proposed diagnosis, treatment need (yes/no), SPT need (yes/no), and prognostic assessment, were analyzed with McNemar’s test, with odds ratios (ORs) and 95% confidence intervals (CIs) calculated from the discordant pairs. The sign test was also used to determine whether there was a difference in the direction of change in prognosis in a positive or a negative direction (e.g. shifting from tooth loss toward no progression or vice versa). Numerical, discrete variables, such as the number of treatment sessions, were analyzed with a paired t-test. All analyses were done using the Statistical Package for the Social Sciences (PC version 27; Armonk, New York, USA). Level of significance was set at *p* < 0.05.

## Results

Of the 174 RDHs enrolled in the study, 82% (*n* = 142) completed the questionnaire on both occasions. This corresponds to 4.3% of RDHs in the Swedish Dental Hygienist Association and 3.3% of the total number of RDHs in Sweden. The 142 participants comprised 92.3% females (mean age 44 years [range 24–68], mean professional experience 14 years [range 1–45]). Data supplied by the Swedish National Board of Welfare confirmed that the gender and age distribution of the study cohort were similar to that of licensed dental hygienists in Sweden overall [18]. Additionally, participants from both the private and public sectors and from different parts of Sweden were included.

### Diagnostic assessment

**Figure 2:**
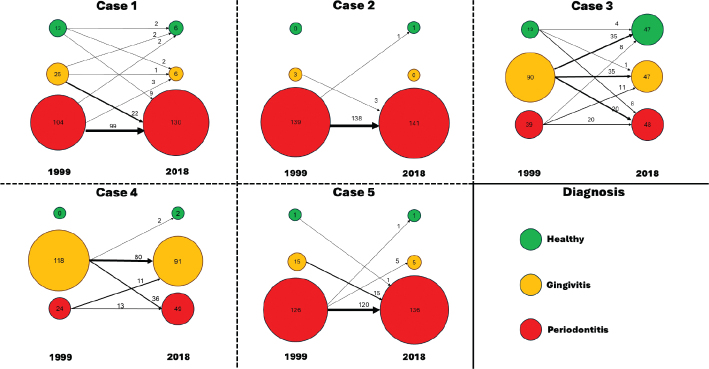
Number of registered dental hygienists (n = 142) and their proposed diagnoses using either the 1999 or the 2018 classification for each of the five cases. Changes are significant (p < 0.05) in Cases 1, 3, and 4. The arrows represent transitions across time points. Case 1, bone loss of > 33%, negligible inflammation. Case 2, bone loss of > 33%, inflammation. Case 3, slight bone loss, negligible inflammation. Case 4, slight bone loss, inflammation. Case 5, bone loss of < 33%, inflammation. (See Figure 1 for more detailed descriptions of the cases).

Differences between the proposed diagnoses were observed in three of the five cases (1, 3, 4), when the different classification systems were applied (Figure 2, Table 2). In Case 1, periodontitis was the predominant proposed diagnosis when applying the 1999 classification. This became even more pronounced when the 2018 classification was used since a substantial number (*n* = 22) of the participants changed their proposed diagnosis from gingivitis (1999) to periodontitis (2018). In Case 4, gingivitis was the predominant proposed diagnosis when applying the 1999 classification; however, this pattern was less clear when the 2018 classification was used. A high number (*n* = 36) of participants changed their proposed diagnosis from gingivitis (1999) to periodontitis (2018). In Case 3, participants shifted from proposing gingivitis (1999) to proposing health (*n* = 35) and periodontitis (*n* = 20) to a greater extent and gingivitis to a lesser extent (*n* = 35) when the 2018 classification was used. In the remaining cases, no significant changes in proposed diagnoses were observed (Figure 2).

**Table 2:** Percentage of participating registered dental hygienists assigning diagnoses and treatment decisions for five cases using the 1999 and 2018 periodontal classifications.

Case	Classification	Case assessment (*N* = 142)					
		Healthy (%)	Gingivitis (%)	Periodontitis(%)	Treatment need (%)	Mean no. of treatment sessions (SD)	Need for SPT (%)
1	1999	9.2	17.6	73.2	46.5	1.18 (1.93)	59.9
	2018	4.2*	4.2*	91.5*	45.1*	0.96 (1.74)	53.5
2	1999	0	2.1	97.9	100	4.56 (2.50)	98.6
	2018	0.7	0	99.3	100	3.75* (1.95)	97.9
3	1999	9.2	63.4	27.5	54.9	1.29 (2.27)	36.6
	2018	33.1*	33.1*	33.8*	35.9*	0.86* (1.86)	23.2*
4	1999	0	83.1	16.9	83.1	2.30 (2.23)	63.4
	2018	1.4*	64.1*	34.5*	83.1*	1.96* (1.92)	69
5	1999	0.7	10.6	88.7	99.3	3.75 (2.36)	94.4
	2018	0.7	3.5	95.8	99.3	3.23* (2.03)	90.1

SD: standard deviation; SPT: supportive periodontal therapy.

The mean number of proposed treatment sessions (± SD) and the percentage of participants proposing are also shown.

* Significant difference (*p* < 0.05) between the 1999 and 2018 classifications for each diagnosis, treatment need, mean number of treatment sessions, and SPT.

Case 1, bone loss of > 33%, negligible inflammation.

Case 2, bone loss of > 33%, inflammation.

Case 3, slight bone loss, negligible inflammation.

Case 4, slight bone loss, inflammation.

Case 5, bone loss of < 33%, inflammation.

(see Figure 1 for more detailed descriptions of the cases).

### Treatment

Only in Case 3 did fewer participants (35) identify a need for treatment using the 2018 classification compared with using the 1999 classification. Conversely, 18 participants changed in the opposite direction (from no treatment to treatment) (OR = 0.39, 95% CI: 0.18–0.78, *p* < 0.05). Most participants assessed patients with clear periodontal inflammation, that is, Cases 2, 4, and 5 (Table 2), as having a need for periodontal treatment regardless of the classification used. They proposed at least one treatment session comprising one, or a combination, of the following options: disease information, oral hygiene instruction, oral hygiene follow-up, polishing, and supra- and subgingival scaling. Regarding the patient cases with minimal gingival inflammation (cases 1 and 3), the same treatment measures were proposed, but to a lesser degree (fewer treatment sessions) regardless of the classification used. The number of treatment sessions, however, decreased, with participants suggesting significantly fewer sessions when using the 2018 classification in all cases except Case 1 (*p* < 0.05) (Table 2). The only difference in proposed supportive treatment between the classifications occurred in Case 3, where fewer participants (*p* <0.05) suggested SPT when the 2018 classification was used as the basis for diagnosis. In this case, 31 participants changed from proposing SPT to not proposing SPT, whereas 12 changed in the opposite direction (OR = 0.51, 95% CI: 0.27–0.93, *p* < 0.05).

### Prognostic assessment

Independent of the classification used, the majority of the participants expected greater bone loss and tooth loss in patient cases with a clear history of bone loss (cases 1, 2, and 5) (Table 3) if no treatment were provided during the following 10 years. A significant difference occurred in Case 2, where participants (*n* = 23) expected less tooth loss using the 2018 classification compared with 11 participants who suggested the opposite (*p* < 0.05). Using the same classification, the majority expected Cases 3 and 4 to remain unchanged, that is, retain their periodontal status, or at most, experience more inflammation but without further bone loss (Table 3). Overall, participants gave a more positive prognostic assessment when using the 2018 classification in all cases except Case 5 compared with the 1999 classification. In cases 1–4, the prognosis mostly changed from less positive to more positive (e.g. from tooth loss to further bone loss only) (Table 3).

**Table 3:** Values represent the percentage responses (registered dental hygienists, N = 142) for each long-term prognosis and patient case using the 1999 and the 2018 periodontal classifications.

Case	System	Long-term prognosis (%)				Change in prognosis in 2018 (%)		
		Unchanged	More inflammation, no bone loss	Greater bone loss	Tooth loss	Negative	Unchanged	Positive
1	1999	26.8	14.1	54.2	4.9	-	-	-
	2018	33.8	14.8	50	1.4	15.5	55.6	28.9*
2	1999	0	1.4	66.9	31.7	-	-	-
	2018	0*	4.2*	72.5*	23.2*	7.7	73.2	19*
3	1999	39.4	33.1	26.8	0.7	-	-	-
	2018	49.3	33.9	18.3	0.7	18.3	48.6	33.1*
4	1999	9.2	51.4	39.4	0	-	-	-
	2018	10.6	57.5	29.6	0	16.2	54.9	28.9*
5	1999	0.7	16.9	73.2	7	-	-	-
	2018	2.8	14.1	79.6	3.5	12.7	69.7	17.6

A change in prognosis using the 2018 criteria for the same patient case was calculated as a percentage.

* Significant difference (*p* < 0.05) between the 1999 and 2018 classifications for each long-term prognosis and change in prognosis between the 1999 and 2018 classifications.

*Negative* change, a shift from a better to a worse prognosis (e.g. from *unchanged* toward *tooth loss* when moving from left to right across the prognosis columns, from using the 1999 classification to using the 2018 classification).

*Positive* change, a shift from a worse to a better prognosis (e.g. from *tooth loss* toward *unchanged*).

*Unchanged:* the prognosis remained the same on both questionnaires.

## Discussion

To the best of our knowledge, this is the first study to investigate the effect of the 2018 AAP/EFP periodontal classification on diagnostic assessments, treatment strategies, and long-term prognostic assessments in general dental care.

The study employed a quasi-experimental within-subject pre- and post-test design, including an educational component related to the 2018 classification. This education was considered necessary to ensure that participants had a sufficient understanding of the updated classification prior to the post-test. The study did not evaluate the educational component itself. Although this design is not methodologically optimal, a randomized controlled trial could have provided stronger internal validity, it was considered appropriate given the study`s objective.

Because all assessments were conducted according to system-specific classification criteria, it is unlikely that participants’ recognition of the cases or recall of previous assessments using alternative criteria influenced the results. Accordingly, recall bias and carry-over effects are not considered major concerns in this context.

No education was provided regarding the 1999 classification system, as it was considered that all participants had been introduced to and trained in it during their undergraduate education and had also used it in clinical practice and during their studies, although for some participants this experience was limited. Therefore, a brief description of this classification was considered sufficient.

To ensure clinical relevance, the patient cases were selected to reflect the variation and severity commonly encountered in the population [14] and within general dentistry.

### Diagnostic assessment

Previous research indicates that patients with generalized severe chronic periodontitis according to the 1999 classification were largely reclassified into comparable categories such as stage III or IV under the 2018 classification [19–21]. In the present study, the proposed diagnosis for cases with clear clinical signs of ongoing periodontitis (Cases 2 and 5) remained unchanged under the 2018 classification, confirming that evident periodontitis is consistently identified across both systems.

In three of the five cases (1, 3, and 4), significant shifts in diagnosis occurred among the study participants when they used 2018 instead of the 1999 classification. Case 1 had clear evidence of bone loss attributed to periodontitis but few bleeding sites. Because the 2018 classification states that a patient with periodontitis cannot revert to being classified as a non-periodontitis patient even though the condition is stable (BOP < 10%), this could explain the increased proposal frequency of periodontitis as a diagnosis. Surprisingly, a considerable number of participants shifted their proposed diagnosis from gingivitis to periodontitis in Cases 3 and 4 as well. This raises the question of why these participants diagnosed periodontitis in cases with no evident bone loss attributed to periodontitis (Figure 2). One reason might be that the participants directly classified these cases as stage I periodontitis because of the presence of PPDs of 4 mm, which is a complexity factor accounted for in the staging process of determining periodontitis. Thus, they may not have initially considered the case definition for periodontitis. According to the 2017 World Workshop, periodontitis should only be diagnosed when interdental CAL is present at ≥2 non‑adjacent teeth, or when buccal/oral CAL ≥3 mm with pocketing >3 mm at ≥2 teeth is present, provided the attachment loss is not due to other causes [22]. Furthermore, the distinction between periodontal health, gingivitis, and periodontitis depends on the interpretation of clinical findings as well. In Cases 3 and 4, variability in how probing depths of 4 mm and bleeding on probing were interpreted may have contributed to differences in proposed diagnosis. For example, if a probing depth of 4 mm is considered a true periodontal pocket, then the patient would not fulfil the criteria for periodontal health. On the other hand, if the pocket is presumed to be a pseudo-pocket, the patient could be considered healthy or having gingivitis [2]. These differences underscore how interpretation of clinical findings can influence diagnostic outcomes. Previous research highlights that distinguishing gingivitis from Stage I periodontitis is challenging due to overlapping clinical characteristics [21, 23, 24]. On the other hand, approximately one third of participants (Figure 2) shifted their diagnostic assessments from periodontitis and gingivitis (1999) to healthy (2018) in Case 3. This indicates that health could be identified if the classification is used as intended.

Since these cases (3 and 4) could be considered borderline, substantial variation in diagnostic outcomes is both expected and evident when applying the 1999 and the 2018 classifications (Figure 2). However, diagnostic variability is a well-recognized challenge in both dentistry and medicine and can be attributed to multiple factors [25–27]. Furthermore, previous studies evaluating the application of the 2018 classification have reported only moderate levels of diagnostic agreement [11, 28, 29].

### Treatment

Many studies have evaluated the 1999 and 2018 classifications, but most have focused on gold standard comparisons or inter-rater reliability, rather than the effect of classification systems on treatment planning. The effect on treatment is important since one of the purposes of the classification is to provide the most appropriate and timely patient care possible. Using the 2018 classification, participants recommended fewer treatment sessions in four out of five patient cases, including Case 2, the most advanced case. The change in proposed diagnosis, from diseased to healthy, could have influenced participants to recommend fewer treatment sessions for Case 3. However, other factors likely played a role in the remaining cases (2, 4, and 5), such as the participants’ improved understanding of the broad spectrum of severity stages in periodontitis as well as the inclusion of risk of disease progression as part of the grading system in the 2018 classification, which is an important part in the treatment decision-making process [30]. The revised and expanded classification added descriptions of extent, severity, complexity, and risk of future attachment loss, which were missing or defined more broadly in the previous classification [31]. The revised classification (2018) includes risk of periodontitis progression partly modelled on the risk assessment model proposed by Lang and Tonetti [32]. Hence, clinicians need to consider multiple risk factors, both systemic and local, during treatment planning and determining risk of progression in periodontitis patients. Therefore, considering the overall clinical picture of the cases with bone loss attributed to periodontitis (Cases 1, 2, and 5) in the questionnaire, these cases could be considered to have a low to moderate risk of disease progression. The more positive assessment of the risk for progression might have resulted in the cases being assigned a less extensive treatment. Furthermore, in Case 3, a high number of participants changed their decision from proposing SPT to not proposing SPT. Since only cases with periodontitis were considered to need regular supportive periodontal therapy, this shift likely reflects the increased number of participants who diagnosed the case as healthy.

Although the number of recommended treatment sessions decreased in four out of five cases, some participants still suggested periodontal treatment in cases with a clinically healthy periodontal status (Case 3). This is consistent with other studies [8, 33], which found that even healthy patients were considered to need periodontal treatment, suggesting that an individually tailored approach is not always implemented. While the latest classification appears to have eased this issue to some extent, our observation suggests that overtreatment of clinically healthy patients may remain an issue, as two-thirds of the participants in this study assigned a diagnosis of gingivitis or periodontitis in this particular case.

### Prognostic assessment

Even though most of the cases were assessed to be more diseased when applying the 2018 classification, participants generally made more positive prognostic assessments. This can be attributed to a more clearly definition of the risk of disease progression through grading. This may have affected the assessment of the cases in this study since they were assessed as low risk for progression because of their clinical and anamnestic data. Even in cases of severe periodontitis (Case 1), the risk of disease progression may be low to moderate if the patient is well controlled.

### Strengths and limitations

Although participation was voluntary and the enrolment rate was relatively low (142 out of approximately 3,300 invited RDHs), the study sample was comparable to the target population in terms of key characteristics, including age, professional experience, and workplace. This similarity supports the representativeness of the sample and strengthens the external validity of the findings. However, the possibility of self-selection bias cannot be fully excluded. Participants who chose to enrol in the study may differ from nonparticipants in unobserved ways, such as motivation, interest in the 2018 classification, or engagement in professional development activities. These factors may influence the results and limit the extent to which the findings can be generalized. In addition, the absence of randomization and a control group limits the ability to draw causal inferences. Another limitation is the fictitious nature of the patient cases. The questionnaire captures self-reported assessments, which may not fully correspond to those that would have been made in routine clinical practice. Furthermore, although the approach in this study allows for standardized case-based assessments, the single-group pretest-posttest design makes it difficult to fully separate the effects of the 2018 classification from those of the training required to use it. Finally, as all participants were working in Sweden, and treatment practices may vary between countries, the generalizability of the findings to other settings may be limited.

## Conclusion

In cases without clear attachment loss, the 2018 classification resulted in greater variation in proposed diagnoses compared with the 1999 classification, with different dental hygienists diagnosing the same case as healthy, having gingivitis, or having periodontitis. This highlights a potential risk of overdiagnosis in borderline cases and underscores challenges in the clinical application of the 2018 classification, emphasizing the need for clearer guidance and calibration. Nevertheless, the 2018 classification appeared to be at least as effective as the 1999 classification in identifying patients with periodontitis and was associated with fewer proposed treatments and more optimistic prognoses, suggesting a potentially more individualized approach. Further studies using real-world clinical data are needed to validate these findings.

## Supplementary Material









## Data Availability

All data supporting the findings of this study are available from the corresponding author upon reasonable request.

## Geolocation information

This nationwide study was based on a sample of registered dental hygienists that was nationally representative with respect to gender and age. The geographic center of Sweden is located at approximately 62°N, 15°E.
