# Supplementary material for: Impact of the 2018 periodontal classification on diagnosis, treatment strategies, and assessment of prognosis by dental hygienists in Swedish general dental care
Source: Acta Odontol Scand. 2026 Jul 31;85:46574. doi: 10.2340/aos.v85.46574 (PMC13440219; doi:10.2340/aos.v85.46574)
Supplement: Supplementary file 2 [file AOS-85-46574-s2.pdf]

Klassifikationer av parodontala tillstånd – Plackinducerad Gingivit och parodontit

BAKGRUND

Klassifikationer av parodontala tillstånd har under de senaste 30 åren utvecklats och modifierats allt eftersom nya forskningsrön har trätt fram. Detta i syfte att möjliggöra för behandlare att utföra korrekt diagnostik och behandling av patienter med parodontal sjukdom (1). Den senaste klassifikationen av parodontala tillstånd togs fram under år 2017 och har därefter gjorts tillgänglig för omvärlden.

Principiella förändringar från tidigare klassifikationer

Den främsta förändringen i den senaste klassificeringen är att man har definierat vad det innebär att vara parodontalt frisk samt när sjukdomen övergår till plackinducerad gingivit. Vidare har man, mer exakt, definierat vad som karaktäriserar en parodontitpatient samtidigt som man har slagit ihop sjukdomsformerna aggressiv och kronisk parodontit till en kategori. Dessutom har man utvecklat ett multidimensionellt system för att klassificera patienter med parodontit i enlighet med hur allvarlig sjukdomen är (stadium) och hur stor risk för progression det föreligger (grad) (1).

Parodontal hälsa

En parodontal frisk patient kan beskrivas som en patient som saknar tecken på fästeförlust och inflammation i tandens upphängningsapparat, d.v.s. avsaknad av tecken som tyder på sjukdom. Det innebär att patienten ska ha ett intakt parodontium utan tecken på fäste- och/eller benförlust. Ett intakt parodontalt fäste innebär att bennivån i förhållande till cement-emalj gränsen ligger mellan 1 till 3 mm i hela bettet. Överskrids denna så kan man fastställa att det föreligger en fästeförlust av parodontiet (2).

Inflammation i parodontiet upptäcks bäst genom blödning vid sondering (BOP), vilket innebär att man sonderar med lätt tryck till botten av en tandköttsficka och avvaktar 15 sekunder för att se om blödning har provocerats (3). Sonderar man samtliga ytor i hela bettet och får en procentsats av antalet blödande ytor i förhållande till ytor man har sonderat så kan man avgöra om patienten är frisk eller sjuk. Ligger denna procentsats under 10 % (mindre än 10 % blödande ytor) och inga fickdjup över 3mm registreras (förutom vid pseudofickor) så kan man beskriva tillståndet som friskt (Tabell 1) (2).

Reducerat parodontium – icke-parodontit patient

Det förekommer att patienter har fästeförlust av parodontiet utan att ha en parodontal sjukdom, t.ex. skador orsakade av tandborstning. För denna patient gäller samma kriterier som ovan förutom att man accepterar att fästeförlusten existerar, dock att den inte är orsakad av parodontit (Tabell 2) (2).

Tabell 1

Intakt parodontium	Frisk	Gingivit
Klinisk fästeförlust	Nej	Nej
Sonderat fickdjup† (pseudofickor exkluderade)*	≤3 mm	≤3 mm
Blödning vid sondering†	<10%	≥ 10%
Benförlust på röntgen	Nej	Nej

†Sondtryck 0,2 – 0,25 N
*Pseudoficka kan även kallas för en falsk tandköttsficka vilket innebär att fickdjupet ökar enbart p.g.a. svullnad av vävnaden, ingen fästeförlust förekommer.

Tabell 2

Reducerat parodontium (icke-parodontit patient)	Frisk	Gingivit
Klinisk fästeförlust	Ja	Ja
Sonderat fickdjup† (pseudofickor exkluderade)*	≤3 mm	≤3 mm
Blödning vid sondering†	<10%	≥ 10%
Benförlust på röntgen	Möjlig	Möjlig

†Sondtryck 0,2 – 0,25 N
*Pseudoficka kan även kallas för en falsk tandköttsficka vilket innebär att fickdjupet ökar enbart p.g.a. svullnad av vävnaden, ingen fästeförlust förekommer.

Plackinducerad gingivit

Plackinducerad gingivit karaktäriseras av kliniska tecken på inflammation i form av rodnad, svullnad och blödning vid lätt sondering. Denna inflammation är orsakad av bakteriellt plack (biofilm) och upphör efter att plack har avlägsnats. Vidare, för att en patient ska få diagnosen plackinducerad gingivit får det inte finnas fästeförlust som är orsakat av parodontit. Antal blödande ytor är lika med eller överstiger 10 % och fickdjupen överstiger inte 3 mm (förutom vid pseudofickor) (Tabell 1) (2).

Dessutom kan plackinducerad gingivit klassificeras enligt dess utbredning, nämligen som lokal eller generell. Lokal utbredning innebär att antal blödande ytor sträcker sig från 10 till 30 % medan generell utbredning innebär att antal blödande ytor överskrider 30 % (2).

Reducerat parodontium – icke-parodontit patient

Som tidigare nämnt förekommer skador i form av fästeförlust som inte är orsakade av parodontit. Vid dessa situationer gäller samma kriterier som ovan förutom att man accepterar att fästeförlusten existerar, dock att den inte är orsakad av parodontit (Tabell 2) (2).

Parodontit

Definition av parodontitpatient

Ett klassifikationssystem bör underlätta identifiering, behandling och förebyggande insatser av parodontit hos enskilda patienter. Därför bör man använda klassifikationen för att:

1. Identifiera patienten som en parodontitpatient.
2. Identifiera den specifika formen av parodontit.*
3. Beskriva den kliniska bilden och andra element som påverkar det kliniska omhändertagandet, prognosen och potential påverkan på den orala och systemiska hälsan.

*I den nya klassifikationen finns det tre former av parodontit: Parodontit, Nekrotiserande parodontit och parodontit som en direkt manifestation av systemisk sjukdom. De två sistnämnda formerna avhandlas inte i detta faktablad.

För att en patient ska identifieras som en parodontitpatient behöver den ha approximal fästeförlust (oavsett magnitud) kring två, icke-intilliggande, tänder eller buckal eller oral fästeförlust ≥3 mm med fickdjup >3 mm vid ≥2 tänder. Vidare får inte denna fästeförlust tillskrivas andra tillstånd som t.ex. gingival retraktion p.g.a. trauma, rotkaries, visdomstandens position eller extraktion och dess påverkan på andra molarens distala yta, endodontisk lesion som sprider sig genom det marginala parodontiet eller vertikala rotfrakturer (4).

När patienten är identifierad som parodontitpatient skall den klassificeras enligt sjukdomssvårighet, behandlingskomplexitet, utbredning, tidigare progressionshastighet och risk för framtida progression (5).

Stadium

Det finns fyra stadier som en patient kan placeras i beroende på sjukdomens och den förväntade behandlingens svårighet. Dessa stadier betecknas som siffror från 1 till 4 där det fjärde stadiet är den svåraste (Bild 1).

Man utgår från den värst drabbade tanden i bettet och bedömer först fästeförlusten kring denna, antingen genom klinisk mätning av fästeförlusten eller genom att granska benförlusten på röntgenbilder. Därutöver bedömer man om tandförluster har skett i bettet p.g.a. parodontit, i denna bedömning ingår även tänder som planeras att extraheras p.g.a.

parodontit. Tillsammans utgör dessa faktorer en grund för bedömning i vilket stadium patienten hamnar.

Därefter kontrolleras faktorer som avgör hur svår patienten blir att behandla, t.ex. fickdjup, furkationsinvolvering, förekomsten av vertikala bendefekter, bettkollaps, tuggfunktion m.m. Dessa faktorer kan höja patienten till en högre stadie men inte sänka den till en lägre. Ett exempel kan vara om patienten har förlorat 20 % av fästet kring den värst drabbade tanden så innebär det att patienten hamnar i stadium 2. Om samma tand har också en furkationsinvolvering grad 2 så höjs patienten till stadium 3.

Således kan man inte backa i stadierna, även efter behandling då faktorer som avgör hur svår behandlingen blir elimineras. Den enda gången man kan sänka patienten till ett lägre stadium är om regeneration av fästet sker, (4-6) dock rekommenderas det att patienten förblir i sitt ursprungsstadium eftersom det ger en bra bild av patientens tidigare erfarenhet av parodontit (7).

**Stadium**		**Stadium I**	**Stadium II**	**Stadium III**	**Stadium IV**
**Svårighetsgrad**	**Klinisk fästeförlust vid den värst drabbade tandytan**	1-2 mm	3-4 mm	≥5 mm	
	**Radiologisk benförlust vid den värst drabbade tandytan**	Inom koronala 1/3 (<15 %)	Inom koronala 1/3 (15-33 %)	Mer än koronala 1/3 av rotlängden (>33 %)	
	**Tandförluster**	Inga tandförluster p.g.a. parodontit		≤4 tänder förlorade p.g.a. parodontit	≥5 tänder förlorade p.g.a. parodontit
**Komplexitet**	**Lokala faktorer**	Max fickdjup ≤4 mm<br>Inga angulära bendefekter ≥3 mm<br>Ingen furkationsinvolvering grad II-III	Max fickdjup ≤5 mm<br>Inga angulära bendefekter ≥3 mm<br>Ingen furkationsinvolvering grad II eller III	Utöver stadium II:<br>Fickdjup ≥6 mm<br>Angulära bendefekter ≥3 mm<br>Furkationsinvolvering grad II-III	Utöver stadium III:<br>I behov av omfattande rehabilitering p.g.a.:<br>· Nedsatt tuggfunktion<br>· Sekundär traumatisk ocklusion (mobilitet grad ≥2)<br>· Bettkollaps<br>· Tandvandring<br>· Glesställning<br>· <10 antagonerande tandpar
**Utbredning**	**För det angivna stadiet**	Lokal (<30 % av tänderna involverade), generell eller molar-incisiv-mönster			

Utbredning

Sjukdomssvårigheten skall även beskrivas i sin utbredning: Lokal, generell och molar-incisiv-mönster. Man utgår från det stadium man har placerat patienten i och bedömer hur många av bettets tänder kan tillskrivas samma stadium, är det mindre än 30 % så blir utbredning lokal, annars är den generell. I mer sällsynta fall är det främst molarer och incisiver som drabbas och utgör då molar-incisiv-mönster (6).

Grad

Det finns tre grader som en patient kan placeras i beroende på sjukdomens progressionshastighet och den förväntade utvecklingen. Dessa grader betecknas med bokstäver från A till C där den sistnämnda beskriver en patient med högst progressionshastighet och/eller risk för progression (Bild 2).

Primärt undersöker man hur progressionen har sett ut under de senaste fem åren. Har man tidigare data från patienten t.ex. tidigare röntgenbilder så kan man avgöra och fästeförlust har skett under dessa fem år och i så fall i vilken utsträckning. Är det en patient man saknar tidigare data från så kan man göra en indirekt bedömning av progressionshastigheten. Detta gör man genom att utgå från den värst drabbade tanden, uppskatta den procentuella benförlusten kring denna tand och dividera detta med patientens ålder. Då får man en kvot som avgör vilken grad av progression patienten har. Utöver detta bedömer man även fallfenotypen vilket innebär att man bedömer patientens plackbelastning i förhållande till patientens fästeförlust.

Därefter bedömer man om patienten har några riskfaktorer som kommer påverka den framtida progressionen av sjukdomen. Man utgår från patientens rökvanor och eventuell sjukdom i diabetes och dess nuvarande kontroll. Dessa faktorer kan höja patienten till en högre grad men inte sänka den till en lägre (4-5).

Grad			Grad A	Grad B	Grad C
Primära kriterier	Direkt bedömning av progression	Fästeförlust över tid	Ingen fästeförlust på 5 år	Fästeförlust <2 mm på 5 år	Fästeförlust ≥2 mm på 5 år
	Indirekt bedömning av progression	Kvot: Benförlust(%)/ålder	<0,25	0,25 - 1,0	>1,0
		Fallfenotyp	Omfattande mängd plack/tandsten med ringa fästeförlust	Mängd plack/tandsten korrelerar med fästeförlusten	Omfattande fästeförlust i förhållande till mängd plack/tandsten; klinisk bild som indikerar snabb progression<br>och/eller<br>tidig sjukdomsdomsdebut, behandlingsresistens efter sedvanlig infektionskontroll
Modifierande faktorer	Riskfaktorer	Rökning	Icke-rökare	Röker <10 cigaretter per dag	Röker ≥10 cigaretter per dag
		Diabetes	Normala blodsockervärden/ Ingen diabetesdiagnos	HbA1c <7,0 % för diabetiker	HbA1c ≥7,0 % för diabetiker

Behandlad parodontitpatient

En lyckad behandling av en parodontitpatient leder till ett förbättrat parodontalt status. Beroende på hur lyckad behandlingen blir kan parodontitpatienten klassificeras som frisk eller med endast gingival inflammation.

För att klassificeras som frisk får endast mindre än 10 % antal blödande tandköttsfickor i bettet registreras. Dessutom får fickdjupen inte överstiga 4 mm (förutom vid pseudofickor) samt att de tandköttsfickor som är 4 mm djupa inte får blöda vid sondering överhuvudtaget.

För att klassificeras som parodontitpatient med gingival inflammation kan patienten ha antal blödande tandköttsfickor som är lika med eller överstiger 10 % men det maximala fickdjupet får endast vara 3mm (tabell 3) (2).

Tabell 2

Behandlad parodontitpatient	Frisk	Gingival inflammation
Klinisk fästeförlust	Ja	Ja
Sonderat fickdjup† (pseudofickor exkluderade)*	≤4 mm¥	≤3 mm
Blödning vid sondering†	<10%	≥ 10%
Benförlust på röntgen	Ja	Ja

†Sondtryck 0,2 – 0,25 N
*Pseudoficka kan även kallas för en falsk tandköttsficka vilket innebär att fickdjupet ökar enbart p.g.a. svullnad av vävnaden, ingen fästeförlust förekommer.
¥Ingen yta med 4 mm djup tandköttsficka som blöder vid sondering får förekomma.

Sammanfattning

Således redogör detta dokument vad som karaktäriserar en frisk patient, en patient med gingivit och en patient med parodontit. Alla patienter som är friska kan övergå till att få diagnosen gingivit, men med insatt behandling och förebyggande åtgärder kan dessa patienter återgå till att vara friska eftersom gingivit är en reversibel sjukdom. Patienter som är mottagliga (känsliga) för parodontit kan utveckla fästeförlust och därmed klassificeras som parodontitpatienter. Denna sjukdom är irreversibel vilket innebär att patienten aldrig kan klassificeras som frisk eller diagnostiseras med gingivit. Vidare kan denna patient klassificeras på basis av dess sjukdomssvårighet (Stadie) och risk för progression (Grad). Efter lyckad behandling kan patienten anses ha ett friskt och stabilt parodontalt status eller ha en viss gingival inflammation. Om behandlingen misslyckas (inte uppnår till kriterier för

friskt eller gingival inflammation på en parodontitpatient) eller recidiverar så klassificeras patientens parodontit som ostabil (Bild 3) (2).

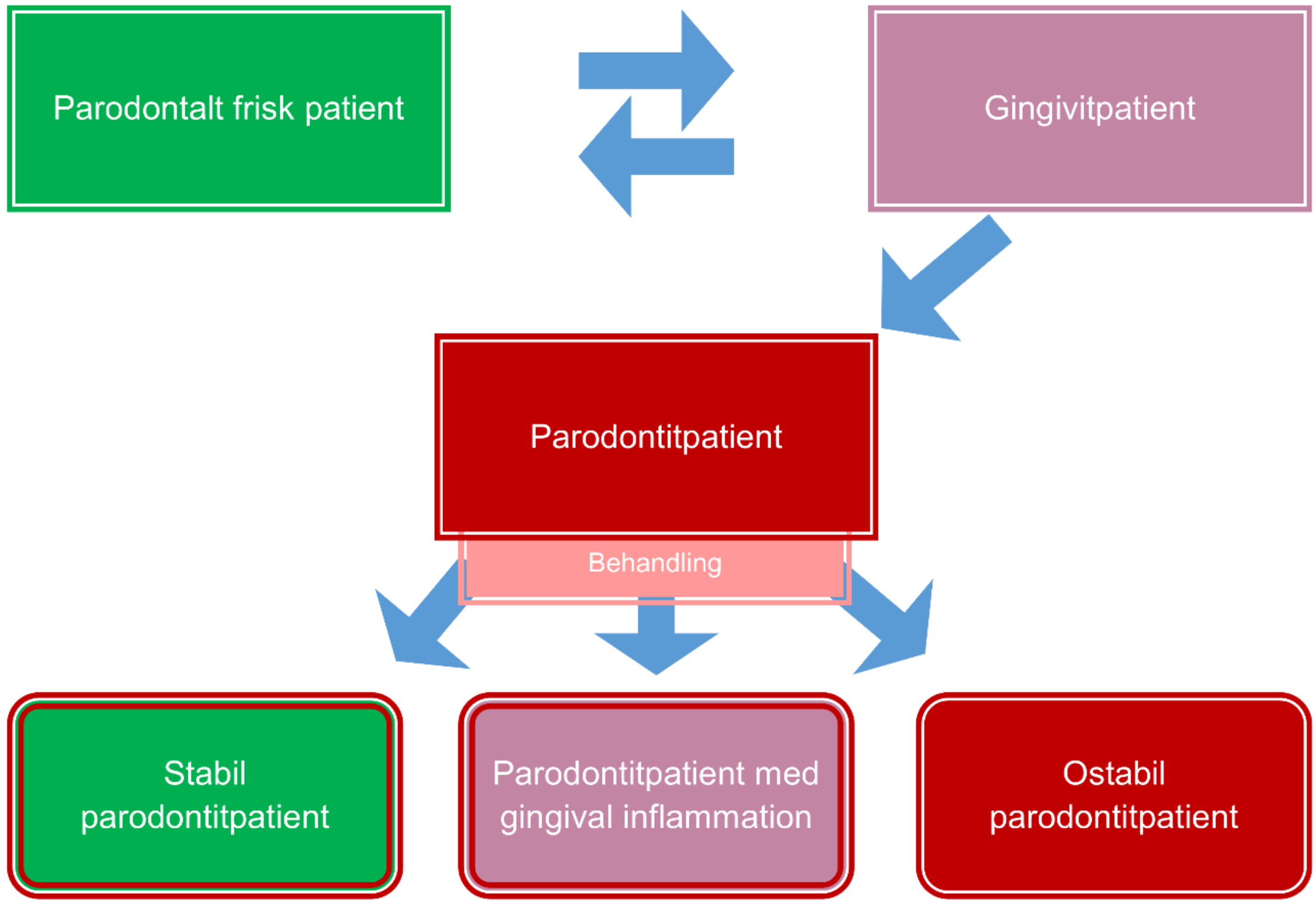

Referenser

1. Caton JG, Armitage G, Berglundh T, Chapple ILC, Jepsen S, Kornman KS, Mealey BL, Papapanou PN, Sanz M, Tonetti MS. A new classification scheme for periodontal and peri-implant diseases and conditions - Introduction and key changes from the 1999 classification. J Clin Periodontol. 2018 Jun;45 Suppl 20:S1-S8. doi: 10.1111/jcpe.12935. PMID: 29926489.
2. Chapple ILC, Mealey BL, Van Dyke TE, Bartold PM, Dommisch H, Eickholz P, Geisinger ML, Genco RJ, Glogauer M, Goldstein M, Griffin TJ, Holmstrup P, Johnson GK, Kapila Y, Lang NP, Meyle J, Murakami S, Plemons J, Romito GA, Shapira L, Tatakis DN, Teughels W, Trombelli L, Walter C, Wimmer G, Xenoudi P, Yoshie H. Periodontal health and gingival diseases and conditions on an intact and a reduced periodontium: Consensus report of workgroup 1 of the 2017 World Workshop on the Classification of Periodontal and Peri-Implant Diseases and Conditions. J Periodontol. 2018 Jun;89 Suppl 1:S74-S84. doi: 10.1002/JPER.17-0719. PMID: 29926944.
3. Ainamo J, Bay I. Problems and proposals for recording gingivitis and plaque. Int Dent J. 1975 Dec;25(4):229-35. PMID: 1058834.
4. Tonetti MS, Greenwell H, Kornman KS. Staging and grading of periodontitis: Framework and proposal of a new classification and case definition. J Periodontol.

2018 Jun;89 Suppl 1:S159-S172. doi: 10.1002/JPER.18-0006. Erratum in: J Periodontol. 2018 Dec;89(12):1475. PMID: 29926952.

5. Papapanou PN, Sanz M, Buduneli N, Dietrich T, Feres M, Fine DH, Flemmig TF, Garcia R, Giannobile WV, Graziani F, Greenwell H, Herrera D, Kao RT, Kebschull M, Kinane DF, Kirkwood KL, Kocher T, Kornman KS, Kumar PS, Loos BG, Machtei E, Meng H, Mombelli A, Needleman I, Offenbacher S, Seymour GJ, Teles R, Tonetti MS. Periodontitis: Consensus report of workgroup 2 of the 2017 World Workshop on the Classification of Periodontal and Peri-Implant Diseases and Conditions. J Periodontol. 2018 Jun;89 Suppl 1:S173-S182. doi: 10.1002/JPER.17-0721. PMID: 29926951.
6. Sanz M, Papapanou PN, Tonetti MS, Greenwell H, Kornman K. Guest Editorial: Clarifications on the use of the new classification of periodontitis. J Clin Periodontol. 2020 Jun;47(6):658-659. doi: 10.1111/jcpe.13286. Epub 2020 Apr 29. PMID: 32274820.
7. Kornman, K. S., & Papapanou, P. N. (2020). Clinical application of the new classification of periodontal diseases: Ground rules, clarifications and “gray zones”. *Journal of periodontology*, *91*(3), 352-360.
