# Supplementary material for: Impact of the 2018 periodontal classification on diagnosis, treatment strategies, and assessment of prognosis by dental hygienists in Swedish general dental care
Source: Acta Odontol Scand. 2026 Jul 31;85:46574. doi: 10.2340/aos.v85.46574 (PMC13440219; doi:10.2340/aos.v85.46574)
Supplement: Supplementary file 3 [file AOS-85-46574-s3.pdf]

Uppslagstabell för diagnosticering av parodontala förhållanden på patientnivå

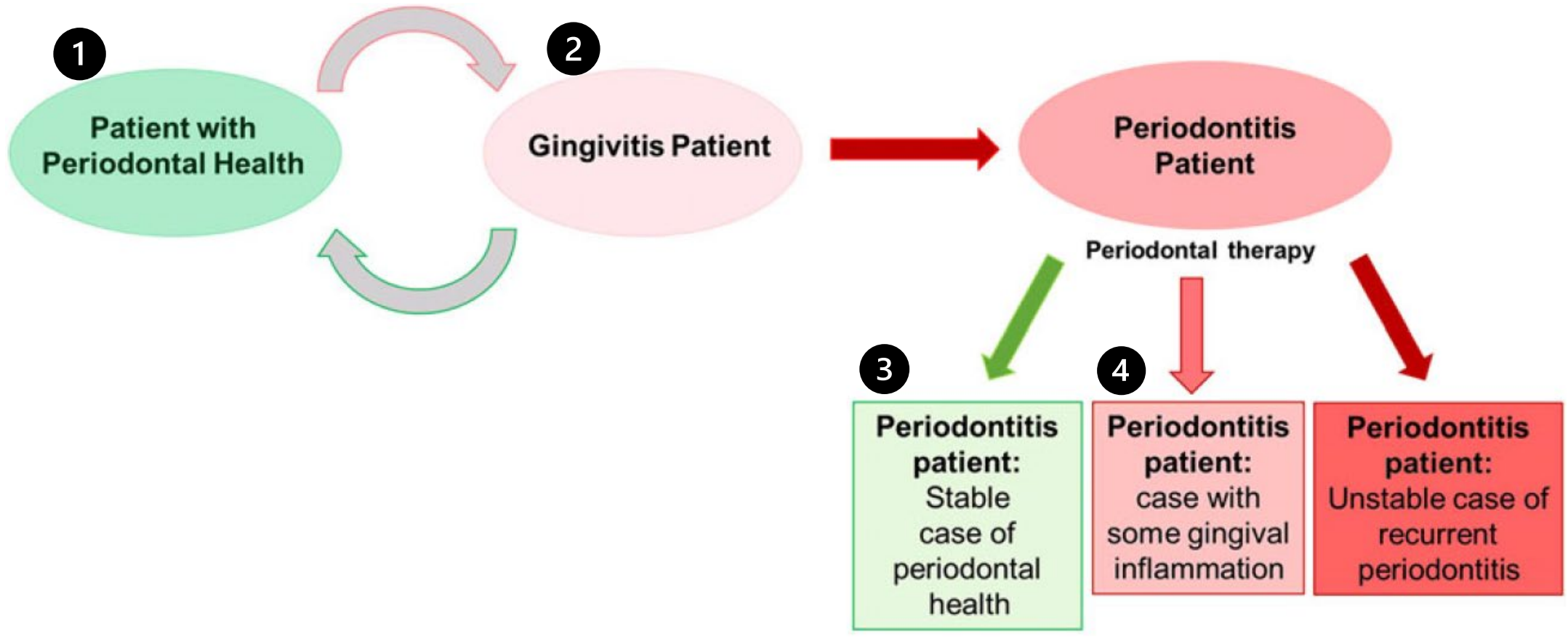

**Intakt parodontium**	**Frisk** ①	**Gingivit** ②
Klinisk fästeförlust	Nej	Nej
Sonderade fickdjup *	≤3 mm	≤3 mm
BOP	<10%	≥10%
Benförlust på röntgen	Nej	Nej

**Reducerat parodontium, icke-parodontit**	**Frisk** ①	**Gingivit** ②
Klinisk fästeförlust	Ja	Ja
Sonderade fickdjup *	≤3 mm	≤3 mm
BOP	<10%	≥10%
Benförlust på röntgen	Möjligt	Möjligt

**Behandlad parodontit med stabila förhållanden**	**Frisk** ③	**Gingivit hos individ med parodontiterfarenhet** ④
Klinisk fästeförlust	Ja	Ja
Sonderade fickdjup *	≤4 mm ^	≤3 mm
BOP	<10%	≥10%
Benförlust på röntgen	Ja	Ja

* Exklusive pseudofickor

^ Inget fickdjup ≥ 4 mm med blödning vid sondering

**Patient med parodontit**	~~**Frisk**~~	**Parodontit**

För patienter med parodontit väljs utbredning, stadium och grad enligt separat uppslagstabell
