# Supplementary material for: Impact of the 2018 periodontal classification on diagnosis, treatment strategies, and assessment of prognosis by dental hygienists in Swedish general dental care
Source: Acta Odontol Scand. 2026 Jul 31;85:46574. doi: 10.2340/aos.v85.46574 (PMC13440219; doi:10.2340/aos.v85.46574)
Supplement: Supplementary file 4 [file AOS-85-46574-s4.pdf]

MALMÖ UNIVERSITET

		Stadium I	Stadium II	Stadium III	Stadium IV
**Svårighetsgrad**	Klinisk fästeförlust vid den sämsta tandytan	1-2 mm	3-4 mm	≥5 mm	
	Radiologisk benförlust	Inom koronala 1/3 (<15 %)	Inom koronala 1/3 (15-33 %)	Mer än koronala 1/3 av rotlängden (>33 %)	
	Tandförluster	Inga tandförluster p.g.a. parodontit		≤4 tänder förlorade p.g.a. parodontit	≥5 tänder förlorade p.g.a. parodontit
**Komplexitet**	Lokala faktorer	Fickdjup ≤4 mm<br><br>Inga angulära bendefekter ≥3 mm<br><br>Ingen furkationsinvolvering grad II-III	Fickdjup ≤5 mm<br><br>Inga angulära bendefekter ≥3 mm<br><br>Ingen furkationsinvolvering grad II eller III	Utöver stadium II:<br><br>Fickdjup ≥6 mm<br><br>Angulära bendefekter ≥3 mm<br><br>Furkationsinvolvering grad II-III	Utöver stadium III:<br><br>I behov av omfattande rehabilitering p.g.a.<br>• nedsatt tuggfunktion<br>• Sekundär traumatisk ocklusion (mobilitet grad ≥2)<br>• Bettkollaps<br>• Tandvandring<br>• Glesställning<br>• <10 antagonerande tandpar
**Utbredning**		Lokal (<30 % av tänderna involverade), generell, eller molar-/incisiv-utbredning			

MALMÖ UNIVERSITET			Grad A	Grad B	Grad C
**Primära Kriterier**	**Direkt bedömning av progressionshastighet**	Fästeförlust över tid	Ingen fästeförlust på 5 år	Fästeförlust <2 mm på 5 år	Fästeförlust ≥2 mm på 5 år
	**Indirekt bedömning av progressionshastighet**	Benförlust Kvot: %/ålder	<0,25	0,25 - 1,0	>1,0
		Fallfenotyp	Omfattande mängd plack/tandsten med ringa fästeförlust	Mängd plack/tandsten korrelerar med fästeförlusten	Omfattande fästeförlust i förhållande till mängd plack/tandsten; klinisk bild som indikerar snabb progression<br><br>och/eller<br><br>tidig sjukdomsdomsdebut, behandlingsresistens efter sedvanlig infektionskontroll.
**Modifierande faktorer**	**Riskfaktorer**	Rökning	Icke-rökare	Röker <10 cigaretter per dag	Röker ≥10 cigaretter per dag
		Diabetes	Normala blodsockervärden/ Ingen diabetesdiagnos	HbA1c <7,0 % för diabetiker	HbA1c ≥7,0 % för diabetiker
